# Unmasking Upper Airway Obstruction: A Case of Tracheobronchial Amyloidosis

**DOI:** 10.1002/rcr2.70357

**Published:** 2025-10-07

**Authors:** Abhir Nainani, Chui Lyn Leong, Vishnu Jeganathan, Siven Seevanayagam, Nicole Goh, Celia Lanteri

**Affiliations:** ^1^ Austin Health Respiratory and Sleep Medicine Melbourne Australia; ^2^ Bendigo Health Respiratory Unit Bendigo Australia; ^3^ Austin Health Thoracic Surgery Melbourne Australia

**Keywords:** positive airway pressure, tracheobronchial amyloidosis, upper airway obstruction

## Abstract

We present a rare case of a 76‐year‐old woman with severe tracheobronchial amyloidosis (TBA) causing airway collapse, in which the predominant underlying mechanism was expiratory central airway collapse (ECAC) secondary to both TBA and severe obstructive sleep apnoea (OSA). CPAP therapy effectively treated severe OSA, which likely contributed to a reduction in ECAC severity and alleviation of breathlessness. This case suggests that positive airway pressure ventilation may represent a valuable adjunctive treatment in patients with TBA exhibiting features of OSA, particularly when endobronchial intervention is contraindicated or not feasible.

## Graphical abstract

Positive airway pressure is frequently utilised in the management of upper airway obstruction and symptomatic ECAC; however, its application in tracheobronchial amyloidosis (TBA) has not been previously described. Our case demonstrates a predominant mechanism of underlying ECAC, attributable to both TBA and severe OSA. CPAP may represent a valuable adjunctive treatment in patients with TBA exhibiting features of OSA, particularly when endobronchial intervention is contraindicated or not feasible.

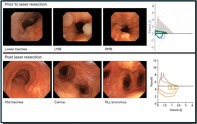

## 1 Introduction

Pulmonary amyloidosis is a rare condition characterised by misfolded proteins that aggregate and deposit in the lungs [1]. It is typically categorised into three subtypes: nodular, diffuse and tracheobronchial amyloidosis (TBA) [1].

TBA occurs in 1% of amyloidosis cases, primarily affecting the central airways, and can lead to obstruction [2]. Management for pulmonary amyloidosis usually involves endobronchial interventions such as localised mechanical debulking, laser irradiation, argon plasma therapy, stenting or balloon dilation [3]. However, options are limited in patients with extensive burden of disease or non‐surgical candidates apart from external beam radiotherapy, which has a delayed effect [3].

The application of positive pressure, a common and effective intervention for upper airway obstruction, has never been reported in the management of TBA. We present a case of TBA with confirmed upper airway obstruction, initially managed with continuous positive airway pressure (CPAP) before undergoing endobronchial laser resection.

## 2 Case Report

**FIGURE 1:**
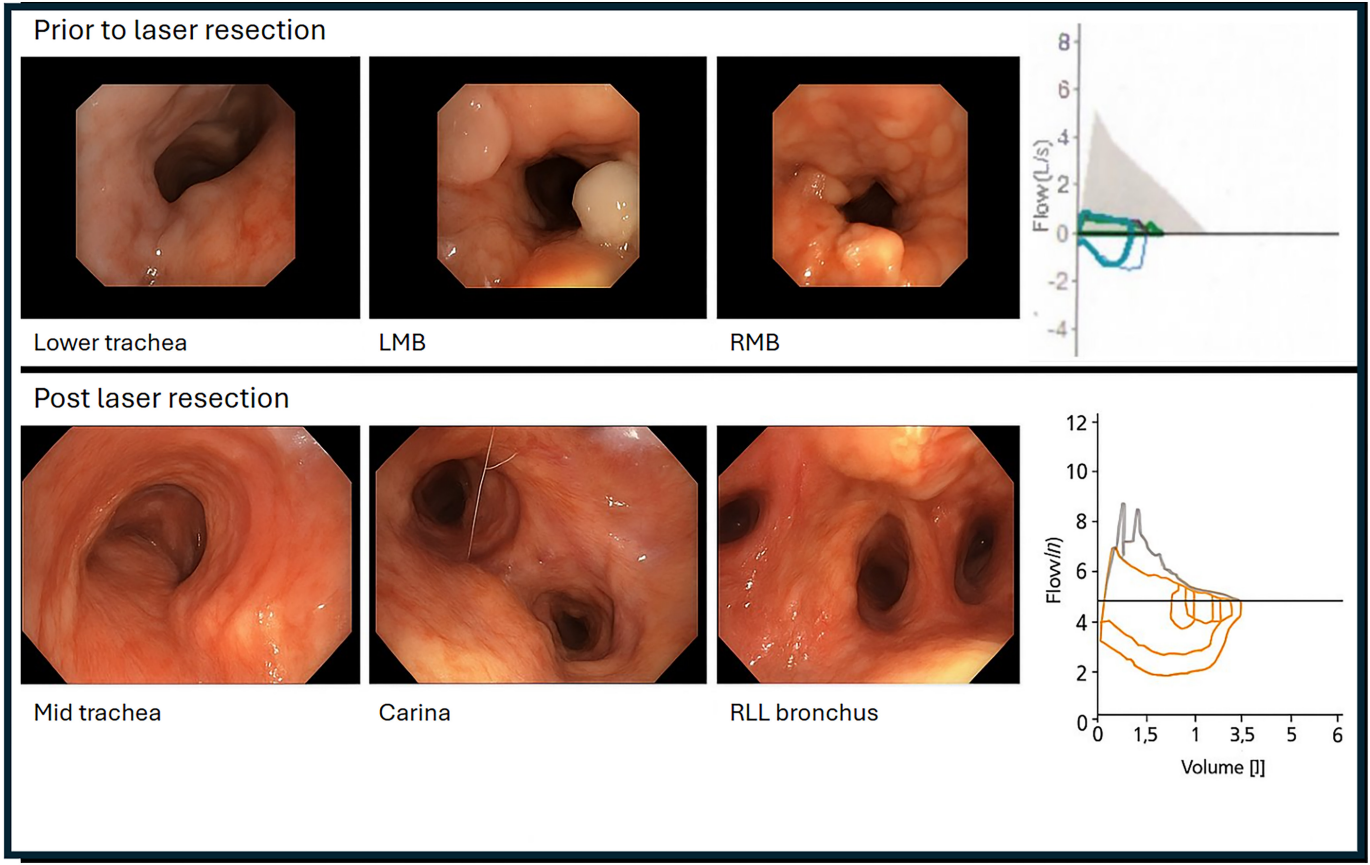
Bronchoscopy views before and after laser resection. Top row: Infiltrative amyloid deposits are seen in the trachea, left main bronchus (LMB), and right main bronchus (RMB) prior to laser resection. The flow‐volume loop demonstrated severe airway obstruction. Bottom row: Follow‐up bronchoscopy at eight months post‐resection shows a marked reduction in amyloid burden with no evidence of recurrence. The flow‐volume loop demonstrates an improvement in the airway obstruction.

**FIGURE 2:**
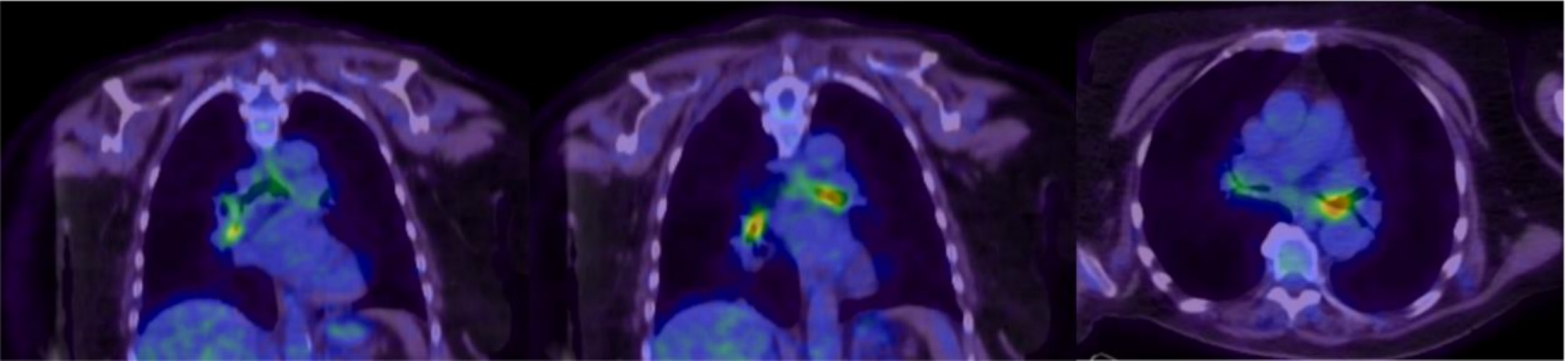
PET scan images. From left to right: Coronal and axial images of FDG‐avid lesions consistent with pulmonary amyloidosis.

A 76‐year‐old woman presented with worsening dyspnoea and stridor despite nebulised saline and corticosteroids. The flow‐volume loop (Figure 1), and spirometry showed severe obstruction FEV1 0.69 L (38% predicted) and reduced FVC 1.37 L (41% predicted), though interpretation was limited by difficulties performing the test due to significant symptom burden. Chest Computer Tomography (CT) revealed concentric airway narrowing and Positron Emission Tomography (PET) showed FDG‐avidity consistent with known diagnosis of TBA (Figure 2). Stenotic lesions along the tracheobronchial tree were identified at bronchoscopy (Figure 1), with more than 50% tracheal collapse during expiration. It was noted that jet ventilation improved dynamic airway collapse during the procedure. Changes of TBA with Congo‐Red birefringence were detected on endobronchial biopsies. Evaluation for systemic amyloidosis, including immunohistochemistry for A amyloid (AA), transthyretin (TTR) amyloid and serum free light chains, were negative and non‐immunospecific. There was no serological or urine evidence of plasma cell dyscrasia (including a normal urine albumin: creatinine ratio).

The patient's disease was initially deemed too extensive and friable for endoscopic endobronchial intervention. In light of observed dynamic lower airway collapse noted during the procedure, the use of positive airway pressure was considered. Polysomnography also demonstrated severe obstructive sleep apnoea (OSA) with an overall AHI of 74.7 events per hour and a trial of both daytime and nocturnal CPAP improved her dyspnoea, although her stridor persisted.

Following 1 month of nocturnal and intermittent daytime CPAP therapy, our patient experienced marked clinical symptom improvement. Her case was reviewed at a multidisciplinary team meeting. It was collectively agreed that she was clinically stable and from an anaesthetics perspective, she was deemed a suitable candidate for repeat assessment for endobronchial laser resection which was considered the most reliable approach for debulking the amyloid deposits. Treatment options were limited as lung transplantation was not deemed appropriate, and chemotherapy was considered but not pursued due to concerns regarding its limited efficacy in reducing amyloid burden. The patient subsequently underwent endobronchial laser resection, which led to complete resolution of her symptoms.

Serial repeat spirometry at least 6 months post‐endobronchial resection demonstrated complete resolution of obstruction (FEV1 1.96 L (102% predicted) and FVC 2.88 L (115% predicted)) and normal spirometry was preserved at 12 months (FEV1 2.09 L (112% predicted) and FVC 2.95 L (121%)). Bronchoscopy at 8 months post‐treatment showed no disease recurrence, and the flow‐volume loop demonstrated an improvement in the expiratory flow plateau (Figure 1). Repeat polysomnography demonstrated persistent severe OSA, albeit an improvement in overall AHI to 53.2 events per hour, with recommendations for nocturnal CPAP use.

## 3 Discussion

TBA is typically caused by localised production of immunoglobulin light chains that can lead to cough, dyspnoea and haemoptysis. Diagnosis is challenging as symptoms are non‐specific and spirometry is often normal. CT features like thickening, calcifications, and narrowing of central airways may facilitate diagnosis, but confirmation requires endobronchial biopsy [1].

Treatment is reserved for symptomatic patients, though evidence is lacking for the optimal approach. Endobronchial interventions are commonly used to relieve airway obstruction in severe cases of TBA, but are technically challenging and do not modify disease progression. External beam radiotherapy can reduce symptoms and lesion size, but effects are delayed. Tracheostomy may be required in severe cases of TBA. The role for immunosuppression and chemotherapy is unclear [3]. Moreover, our patient has clinically severe tracheobronchial amyloidosis, which is a variant of localised AL amyloidosis that is not only rare, but lacks standard treatment protocols or randomised controlled trials to guide management decisions.

Our case provides insight into potential factors contributing to central airway obstruction in TBA. Although airway obstruction is typically linked to tracheal stenosis [1, 4], we speculate that the presence of concurrent severe OSA may have contributed to large negative intrathoracic pressure shifts and the development or exacerbation of Expiratory Central Airway Collapse (ECAC) in an already chronically inflamed airway. Further supporting this diagnosis, the flow‐volume loop showed a flattened expiratory phase, consistent with a variable intra‐thoracic obstruction, and bronchoscopy revealed a more than 50% reduction in tracheal diameter during expiration—a key feature of ECAC [5].

Positive airway pressure is frequently utilised in the management of upper airway obstruction and symptomatic ECAC [5]; however, its application in tracheobronchial amyloidosis (TBA) has not been previously described. In this case, the predominant mechanism underlying airway collapse was ECAC, attributable to both TBA and severe OSA. CPAP therapy effectively treated severe OSA, which likely contributed to a reduction in ECAC severity and alleviation of breathlessness. Our case suggests that positive pressure ventilation may represent a valuable adjunctive non‐invasive treatment option in patients with TBA exhibiting features of OSA and/or ECAC, particularly when endobronchial intervention is contraindicated or not feasible. This is particularly relevant in cases of severe TBA, where endobronchial interventions form the mainstay of treatment to relieve airway obstruction.

## Author Contributions

C.L.L., C.L., V.J. and S.S. were directly involved in this patient's care. C.L.L., C.L. and A.N. conceptualized the project. A.N. and C.L.L. authored the manuscript. C.L.L., C.L., V.J., N.G. and S.S. reviewed the manuscript.

## Consent

The authors declare that written informed consent was obtained for the publication of this manuscript and accompanying images using the consent form provided by the Journal.

## Conflicts of Interest

The authors declare no conflicts of interest.

## Data Availability

The data that support the findings of this study are available from the corresponding author upon reasonable request.
