# Correction to “Unmasking Upper Airway Obstruction: A Case of Tracheobronchial Amyloidosis”

**DOI:** 10.1002/rcr2.70392

**Published:** 2025-11-02

A. Nainani, C. L. Leong, V. Jeganathan, S. Seevanayagam, N. Goh, C. Lanteri, “Unmasking Upper Airway Obstruction: A Case of Tracheobronchial Amyloidosis,” *Respirology Case Reports* 13, no. 10 (2025): e70357, https://doi.org/10.1002/rcr2.70357.

The second flow volume loop in Figure 1 was inaccurate. Below is the correct Figure 1.

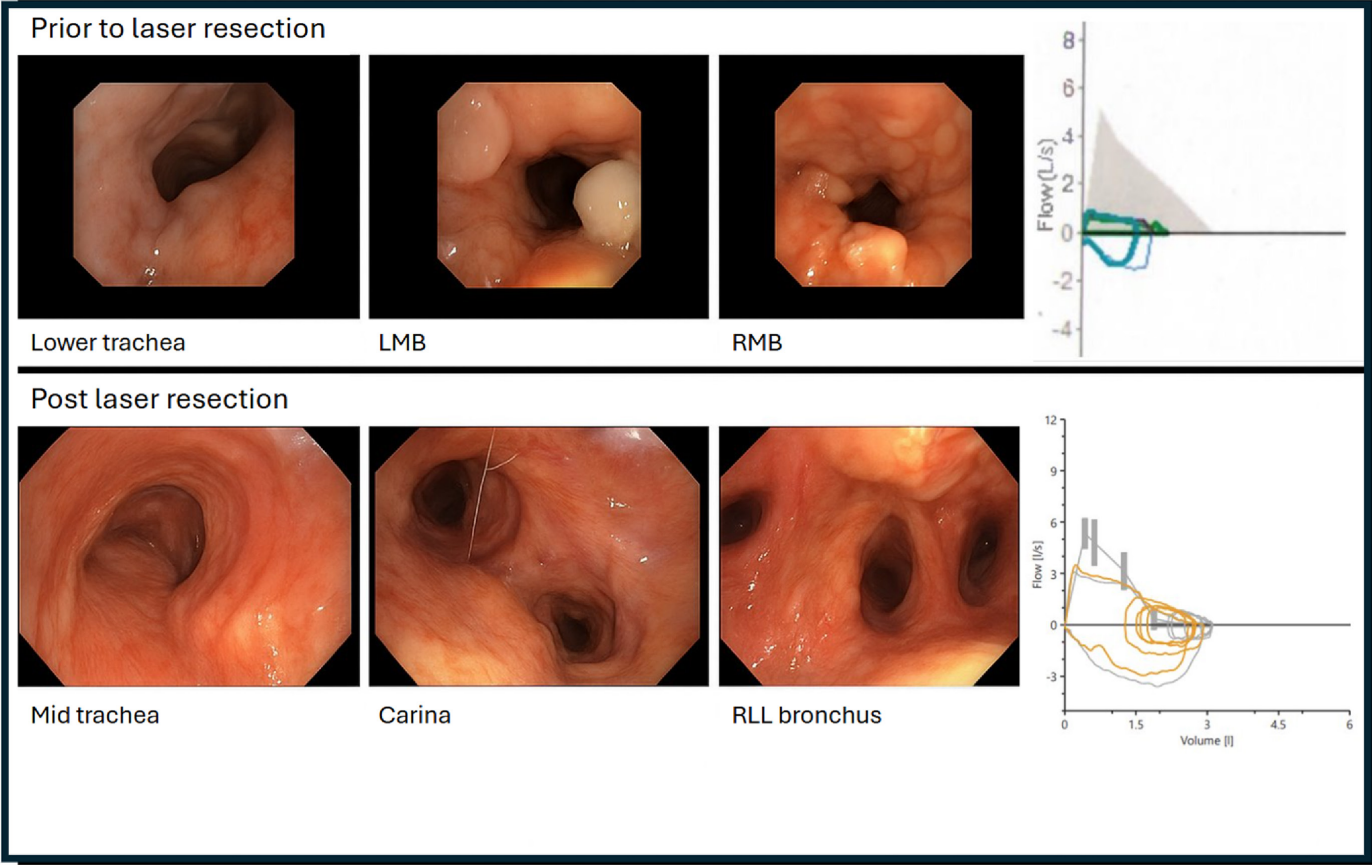

We apologize for this error.

